# Many Minds, One Model: Exploring Decision Making of an Undergraduate Medicine Competency Committee Using the Construct of a Shared Mental Model

**DOI:** 10.5334/pme.1949

**Published:** 2025-08-13

**Authors:** Tim Mickleborough, Glendon R. Tait, Maria Mylopoulos, Kulamakan (Mahan) Kulasegaram

**Affiliations:** 1 Department of Paediatrics, Temerty Faculty of Medicine, University of Toronto, Toronto, Ontario, Canada; 2 The Wilson Centre, Temerty Faculty of Medicine, University of Toronto, Toronto, Ontario, Canada; 3 Wilson Centre, University of Toronto, Temerty Faculty of Medicine, Toronto, Ontario, Canada; 4 Wilson Centre, University Health Network, Canada; 5 Temerty Faculty of Medicine, Associate professor, Department of Family & Community Medicine, University of Toronto, Canada; 6 Learner Assessment and Program Evaluation, Toronto, Ontario, Canada

## Abstract

**Introduction:**

Competency committees (CCs) are considered mandatory in competency-based medical education. There remains insufficient research to guide programs in optimizing the work of CCs especially in the undergraduate context. In order to address this gap, the functioning of an undergraduate CC is examined using the construct of a shared mental model (SMM) to explore factors and context that inform a holistic review of performance.

**Methods:**

A qualitative exploratory study was conducted. Using purposive sampling, 10 members of a Student Progress Committee (SPC) participated in 60-minute, semi-structured interviews (April 2022 to June 2023). An abductive thematic analysis approach generated themes which were then mapped onto a mental model construct. This heuristic helped construct and visualize the inner workings of a SMM as a holistic decision-making process that operates on manipulating multiple data inputs (quantitative and qualitative) in order to generate robust outcomes.

**Results:**

SPC members shared similar expectations of the task at hand while having multiple and conflicting perspectives about inputs important for decision making. Members grappled with what they perceived as a subjective process but agreed that having principles specific to holistic decision making can generate robust outcomes. Diversity of group membership was essential for minimizing member bias and group conformity in decision making.

**Discussion:**

This new understanding of how CCs operate at the undergraduate level can inform the SPC and guide its members in their quality improvement efforts and inform broader program-wide improvement, locally; moreover, it may contribute to the ongoing improvement of CCs in other settings.

## Introduction

Programmatic assessment is a pedagogical approach that shifts emphasis from the decision-making function of assessment to include the value of assessment for learning; an approach well suited, and crucial to competency-based medical education [1,2]. In programmatic assessment, frequent, lower-stakes assessments are chosen to be fit for purpose and are constructively aligned with the curriculum. Data points are then used to provide timely feedback on learner performance and inform coaching and tailored supports. Over longer periods of time, multiple data points, quantitative and qualitative, are aggregated to provide a student performance profile, which when holistically reviewed, inform high-stakes academic decision making such as whether a course or year is satisfactorily completed [1,2]. A key principle of programmatic assessment is that high-stakes decisions are made in a credible and transparent manner using a holistic approach. This is often operationalized through a group process such as a competency committee composed of arm’s length and expert members who can make these decisions [2,3,4].

Competency committees (CCs) have gained importance in postgraduate training programs and are considered mandatory in competency-based medical education by some national and international regulatory bodies [4,5,6,7,8]. National guidelines direct CCs on their structure and function, but there is also autonomy for programs to determine certain aspects such as their composition and size, how they gather and share information, the number of meetings, and methods of reporting [9]. While the function and operation of CCs has been studied for some time, particularly in the postgraduate context, there remains insufficient research to guide programs in optimizing the work of CCs [7,10,11,12]. Given the high-stakes nature of the work, and the need for transparency when making credible decisions, more research is needed to understand how CCs function [9].

### Shared mental models

Drawing primarily from the group decision-making literature [7,9,13,14,15,16,17,18,19], some elements of best practices and guidelines for optimizing CCs have emerged including: 1) a diverse membership in order to generate a range of opinions and possible solutions [3,10,18], and 2) a shared mental model [3,20,21] which would enable members to ‘interpret information consistently, explain findings, and determine actions appropriately for their charge’ [18 p.158]. The value of shared mental models to understand and optimize group processes has particular promise for medical education [19]. Shared mental models are beneficial as they promote team efficiency and functionality. When team members have shared knowledge about the nature of the task and how to complete it, the group functions more cohesively and makes better, and more consistent decisions [19]. The decisions made by CCs determine student progress and group members are accountable to ensure that competent practitioners are entering the profession. A poorly functioning committee could lead to less than optimal outcomes, even legal challenges, thus it is important that group members have a shared mental model to ensure that they make robust decisions that have credibility and are trustworthy [2,21].

While shared mental models have been proposed as integral for optimizing group processes in postgraduate CCs [3,18,19,21,22,23] the undergraduate context remains largely unexamined [24]. Research on postgraduate context has focused on multiple aspects of CCs’ decision-making processes including: reducing bias through a diverse committee membership [3,10]; strengthening validity of milestone evaluations through the incorporation of multiple data points [3,10,21]; robust decision-making through a series of checks and balances [10]; a common understanding of the task at hand requiring that members work from the same set of expectations [19,21]; and open communication for knowledge sharing and making defensible recommendations [19,21,25].

While it is likely many of these recommendations may apply to undergraduate training CCs, this context also creates new issues. It is important to note that the undergraduate context is distinct given that assessment data, both quantitative and qualitative, are more heterogeneous, and designed to assess progress toward competency as an undifferentiated medical doctor [24]. This is in contrast to a primarily workplace-based assessment framework within specialty training programs. In order to begin to address this gap, in this study we examine how CC members construct their understanding of the functioning of an undergraduate CC using qualitative methods. We used the construct of a shared mental model to explore the factors and context that enter a holistic review of performance in the undergraduate setting to determine if there any similarities and differences compared to the postgraduate setting.

## Methods

### Context

The University of Toronto’s Doctor of Medicine (MD) Program implemented programmatic assessment in 2016 as part of the program’s first full curriculum renewal since 1992. The program has about 300 students in each program year. Details of this implementation, including the assessment framework and standards are described elsewhere [26,27]. The Student Progress Committee is charged with making high-stakes decisions about academic performance for the pre-clerkship (Foundations) years at the end of each consecutive course. Our aligned approach to renewal allowed courses to serve not only curricular purposes but also time-based “buckets” of all assessment data for review to inform progress decisions. Within a course, data for consideration includes quantitative and qualitative data which are mapped to our competency framework. Standards are clear for each assessment modality and are non-compensatory. For example, written assessment performance does not negate the requirement for satisfactory professionalism. Standards for written assessment are set in aggregate across a course; hence, learners can be unsatisfactory in some data points but satisfactory overall. Review of data informs not only progress decisions but guides curriculum directors on how to support students’ tailored learning plans, reassessments, and supports.

The Student Progress Committee is composed of 10 voting members most of whom are arm’s-length from direct curricular assessment and student support functions. Most of the current members have been on the Student Progress Committee for at least five years and have developed and iterated an approach for decision making over time. Non-voting members, including course and curriculum leads, act as key informants, presenting performance data and contextual factors that they feel intervened in their performance, informed by their coaching meetings with students. Committee members are experienced in competency-based medical education given this was the dominant paradigm in Canada in postgraduate medical education even prior to its application in undergraduate medical education. Training of members included orientation to the principles of programmatic assessment and our renewed curriculum. Design of committee composition and process was according to best practices [4]. Members were oriented to qualitative, holistic decision making utilizing quantitative and qualitative data. They were introduced to elements that foster reliable and valid approaches to decision making including the value of having a diverse committee and member perspectives, and the importance of critical discourse about decisions and process. Regular debriefing and reflection occurred combined with frequent re-orientation by the Chair to key principles. Expertise development was iterative and tailored to members’ needs.

For feasibility reasons, the Student Progress Committee reviews only students who are flagged as being in difficulty in any portion of the Foundations curriculum (i.e. a student who is meeting standards in all aspects of a program at a given point in time does not get reviewed). Based on a three-year average, the Student Progress Committee conducted 67 detailed holistic reviews of students in difficulty in the Foundations Curriculum (some students are reviewed in more than one course, hence in a year, 56 unique learners were reviewed). For every student reviewed, voting members consider the entirety of the data in the electronic portfolio and probe key informants to understand context and a student’s level of engagement with recommended supports and related outcomes. Members also review the Learner Chart, the electronic portfolio that houses assessment data, and Student Progress Review documents, which articulate students’ reflections on performance and their learning plans. The voting members then discuss, vote, and make recommendations to the Board of Examiners regarding satisfactory progress, partial progress (requires focussed learning and reassessment), or unsatisfactory progress (requires formal remediation).

### Participants

Committee members of the University of Toronto MD Program Student Progress Committee were recruited for the study. Approval for recruitment was provided by the University of Toronto Research Ethics Board. Members who have been on the committee for under a year were excluded because they may lack experience with the committee’s decision-making process to provide insights. Past members were also excluded as their experience may not be as relevant for the committee’s current operation. The chair of the committee (GT) was also excluded from the study. Non-voting members such as course and program directors and members of the University of Toronto’s Office of Learning Affairs were included as informants. We purposively focussed on the voting members for analysis since they were responsible for making decisions regarding student progress; however, non-voting members offered insights into their role as student advocates and providers of high level, confidential data which would be used as inputs into decision making.

Altogether, 8 out of the 10 voting members, and 2 non-voting members agreed to be interviewed for the study. The number of participants (10 in total) reflects the typical make-up of one of the Student Progress Committee’s quarterly meetings and corresponds to the recommended size for CCs [14]. The sample size was felt to be sufficient to hold enough information power to achieve the aim of the study [28,29]. The concept of information power proposes that the larger the information power generated from the sample, the lower the sample size needed. The data had significant information power due to the following key dimensions: a narrow study aim, which was to identify whether the Student Progress Committee had developed a shared mental model for decision making; the specificity of the sample, all participants were members of, or contributed to, the Student Progress Committee; and an established conceptual framework on mental models that informed the analysis while building on existing theory [28].

### Data collection

Voting members of the Student Progress Committee were invited to participate in a 60-minute, semi-structured interview, and were asked questions designed to understand the factors and context that informed their decision making and how they adapted the model over time. Non-voting members were asked questions to provide background regarding their role on the committee as providing insights into the intersection of learner performance with intervening circumstances and engagement with supports, and as advocates for outcomes they anticipate will best serve the learner. Interviews took place virtually which were recorded and then auto transcribed. The transcription was then reviewed by author, TM, for accuracy. Identifying information was removed from the transcripts prior to analysis.

### Data analysis

Interview data was analysed using an abductive thematic analytic approach [30]. This approach was chosen for its flexibility when analyzing qualitative data. It occupies a “middle ground” between inductive and deductive thematic analysis as it engages with both empirical data, providing a rich description of data, and extant theoretical understandings, providing opportunities for theory generation. The approach is a pragmatic one that aims to find the ‘most logical solution and useful explanation for phenomena’ [30, p. 1411]. Observational methods were used in other research on CCs [31], but because of the confidential nature of the meetings, we considered interviews to be a better option for data collection.

Thompson (2022) developed a nuanced approach to abductive thematic analysis based on principles taken from thematic analysis [32] and from abductive research [33]. The first four steps of the analysis mirror that of inductive thematic analysis. Analysis began with transcription and familiarization of data followed by coding. TM independently analyzed the transcripts. After coding the first four interviews, the research team (K.M., M.M., G.T., T.M.) met to develop and iteratively revise codes representing the data. After the first coding meeting, the interview protocol was revised to gain a more fulsome appreciation of how members adapted their approach when faced with certain challenges such as having different opinions or when decisions departed from precedent. The remaining participants were asked questions from the modified protocol. A constant comparative approach was adopted in which new data was compared iteratively with analyzed data to inform the generation of themes.

**Figure 1:**
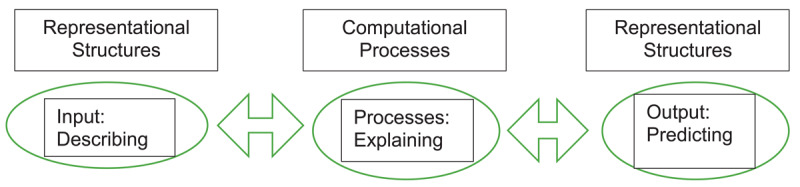
Representation of a mental model. Functions of a mental model represented as inputs, outputs and computational processes.

In an abductive analysis, the next step is to utilize theory to understand the relationships between themes [30] and a mental model framework was instructive in this regard. Mental models allow people to make sense of their world, and they shape how we take action [34]. Mental models have three main functions: to describe the purpose and structure of a system, to explain its functioning by making explicit the nature and relationship of system elements, and to predict future system states [34,35]. They are highly adaptable, internalized belief structures that represent external realities allowing operators of the model to interact effectively with their environment [34,35]. They are also computational processes by which users manipulate model parameters in order to adapt to changing conditions [35]. A representation of the model is shown in Figure 1. The model helped visualize the inner workings of a shared mental model which then became the framework for mapping the coded data. In this construct, the three main functions of the model: representational input and output models (describing and predicting), and the computational, or process module (explaining) became the overarching themes. Sub-themes and related codes generated from the inductive analysis were then mapped onto the framework depending on whether they corresponded to inputs (how members described elements of the system), processes (how members adapted the model by manipulating elements), or outputs (shared outcomes and predictions of decision making) (see Appendix). The data set was then recoded using the theoretically informed structure of the model to confirm conceptual framework and findings. This study received ethical approval from the research ethics board at the University of Toronto (#42105).

## Results

The results show how Student Progress Committee members share similar expectations of the task at hand (the output) while having multiple and often conflicting perceptions about the elements or inputs into the model. This conforms to the conception of an optimal functioning shared mental model: one that generates compatible expectations while allowing for the potential for individual differences [34]. From the abductive analysis, a shared mental model that is unique to the Student Progress Committee was generated reflecting the three functions/overarching themes: how members describe various Student Progress Committee model elements, their explanations of how the model operates, and shared predictions or expectations of the task (Appendix). Below is an overview of each of these functions with a focus on shared and differing aspects of the model and where tensions and paradoxes exist.

### Describing

Holistic decision making is a subjective or contextualized process which requires input of multiple data points including numerical grades and student narratives. In theory, all members agree with this principle, but some expressed discomfort with a process that takes into account aspects that are not ‘quantified or quantifiable’ (P3). Instead, some members prefer an objective approach such as an algorithm allowing them to synthesize data with ‘certainty and consistency’ (P5). The subjective nature of holistic decision making makes some members uncomfortable, but at the same time they realize that subjectivity is inherent to the process. As one member notes, ‘I think… most of our discomfort comes from [the fact] that it feels subjective, but I think…it is supposed to be subjective, and it is supposed to be holistic’ (P6). Members grapple with the subjective nature of the process and reconcile their discomfort by recognizing its importance while being upfront in their approach:

This is not the most objective of committees. If it was…, it would be fully based on [whether] they passed or failed a certain number of assessments…— that would be a truly objective way of doing it…. I think it’s impossible to remove the subjectivity of the members…. I haven’t been able to employ a strategy to remove that, but I think subjectivity is important to the discussion, and being clear about the angle you’re coming from and just being honest about that (P5).

While some members feel more comfortable with the ‘objectivity’ that a numerical-based assessment brings to decision making, one member cautions against this false sense of security while providing some insight on the need for balancing both types of data. In their words:

I think it’s disingenuous to think that if we only look at hard data there will be no bias…. We will always interpret even hard data with our own bias and we will create our own narrative behind [it]…, so without the narrative, each of us gets to build our own narrative, and without discussion, we build our own narratives in our head, and it’s less transparent (P8).

The Student Progress Committee is structured to be diverse. Its members come from various clinical and personal backgrounds, and they have different expertise in medical education including equity, diversity, and inclusion as well as professionalism and remediation. Members agree that having diverse insights into why students struggle is important to inform decision making and promote discussion, but they also acknowledge that processing multiple perspectives is not easy, and it was something that they struggled with in the beginning. As one member notes:

There’s definitely a learning curve and I’d say I’m still learning…. I review the learner charts quite thoroughly before the meetings and that helps me make some of my own decisions before I even hear the presentations coming from the rest of the group,…and then I try to integrate the two at the time. I do the thorough review before because I want to make my own decision before I’m kind of influenced by others (P7).

While a diverse committee can generate multiple insights, incorporating different points of view can reinforce the subjective nature of the process:

We all bring different expertise and it’s really important that we all come up with a different lens, but at the same time that makes it feel like we don’t have an algorithm that were following, so…it feels like we’re just making subjective decisions, but subjectivity is actually part of it. We bring our lens as an expert and we’re going to be getting a subjective opinion based on holistic picture, but it feels very uncomfortable, and it feels like a big burden (P6).

Members are chosen specifically for their subjectivity or unique perspectives, and while this can offer multiple insights, paradoxically, it can also introduce biases that may affect the integrity of the outcome. As one member explains, biases are part and parcel of a diverse representation, but having varied perspectives allows for multiple points of view that in turn improves the integrity of the outcome:

When you’re making decisions about learners in difficulty, you may identify more or less with certain learners, [and] certain learner’s behaviors may trigger you or lead you to make certain conclusions, and as much as we all [have] implicit bias training, and we try to acknowledge our biases, you can never eliminate bias. So the more varied background that you have on the committee, the more people will either speak up in favor or against certain learners, and so you will not always have the whole committee defending one type of learner and penalizing another type of learner (P8).

### Explaining

Members are predisposed regarding the importance of certain elements or inputs into the model, but in terms of the actual processes of decision making, there is more alignment with respect to how they comprehend the meaning and manipulation of different model elements. Foundational to the process of holistic decision making is a number of shared principles including: shifting perspectives, reflecting, listening, deliberating, balancing, and judging. Principles elevate the decision-making process beyond a subjective exercise and during the meeting members are reminded of these principles by the Student Progress Committee chair. One member finds reminders helpful in this regard:

[A] reminder of those principles …and actually [more] knowledge about the principles that the progress committee should be following because it’s new to us…and members of our committee have expertise in it, but not everybody has expertise and when the expertise is not at the forefront of our mind it feels like we’re just making subjective decisions (P6).

One important principle is having the space for negotiation or shared discussion as all members agree that time is needed for deliberation in order to give the process its due diligence. One member sums it up as follows: ‘There’s an appetite for understanding the big picture [that] invites so much questioning and reflecting, and so there’s lots of space made for taking seriously the complexity of the situations that each student faces’ (P4). Another important principle is to be “moved” during decision making and be open to other points of view. One member emphasizes the tensions they experience with this type of negotiation:

I actually think [it’s] a really necessary part of the process…[that] we can be moved in our decisions and that actually helps because then it feels like I’ve challenged myself to really question, “is this what I really think?” Sometimes when I change my mind, at first…it might feel like, “oh, I’m not opinionated enough, I don’t stick to my guns,” but if I reflect on my job, [it’s]… to be moved one way or the other based on evidence … and to me, I think that’s a valuable thing (P6).

Other principles executed in the decision-making process include active listening; an element that one member considers essential for robust decision making. In their words, ‘I mean integrity to include a lot of things, so integrity of the decision-making process includes active listening and deliberation to ensure that you are making decisions that are well informed’ (P8).

In order to interact successfully with the model, users must have knowledge about its operation including knowing how to activate processes to mitigate biases. Members ensure that their biases are kept “in check” by operating key principles of judging and balancing. Student Progress Committee members come from diverse backgrounds so they may hold certain biases when it comes to judging student narratives. As one member notes, the weight committee members give to student narratives ‘comes down to [their] personal backgrounds and how much forgiveness we think should be granted because of the narrative’ (P8). One way to minimize personal or professional biases is balancing narratives with numerical data. One member prefers an approach in which they ‘prioritize the data [first] and then the narrative comes in to inform that’ (P5). They recognize that they need to make judgments as to whether the narrative is compelling enough to offer reassessment and most of the time it is compelling enough to say, ‘this person needs to have…another opportunity.’ However, other times, it is not, so to check their bias they balance their judgement (whether for or against the narrative) against numerical data such as seeing ‘a pattern of challenges with assessments [or] a pattern of not meeting a standard over time’ and that’s when they reflect and think ‘we’re failing-to-fail this person’ (P5).

### Predicting

A shared mental model is effective when operators of the model have compatible expectations of the task at hand, while still allowing for differences with respect to how members describe elements of the model. One member agrees with this interpretation: ‘I think, a shared belief in the decision- making structure, and the voting, that’s important, and a shared value in discussion is important, but I don’t think we have to have shared values and opinions about each learner’ (P8). Members of the Student Progress Committee are in agreement with the expectations of the task: Based on a robust decision-making process, they are able to predict the likelihood of a student’s success if they are given the opportunity to reassess. Thus a “good outcome” for the Student Progress Committee is tied to the integrity of the decision-making process and the expertise of those in the room. As one member explains, ‘a good outcome would be that students are treated fairly by a group who have true expertise in assessment and that we walk away also feeling good about our decision making and not feeling uneasy about our role’ (P6). An agreed upon “good outcome” is promoting and supporting students whom members feel are likely to succeed while at the same time identifying those who are in difficulty. As one member puts it: ‘A good outcome is making sure the decisions we make are robust and that we are promoting “the right” students to succeed, while also identifying those who,… despite their best efforts, despite optimal supports,…may struggle as physicians in practice’ (P5). On the other hand, a “bad outcome” is failing-to-fail students, and this member predicts that the committee may be ‘doing [the students] or the community a disservice by continuing to offer them reassessments’ (P5).

One of the factors considered by members is medicine’s culture of failure-to-fail. However, paradoxically, members could be swayed by students’ extenuating circumstances and feel compelled to offer additional chances. One member explains that when ‘you hear stories [about] why someone might be struggling-- that’s when it’s sometimes harder to be objective. [When] you hear those very heart-wrenching and heart-warming stories--that’s when it gets harder to keep someone behind’ (P7). Another agrees that as educators, it’s harder to fail students and they are tempted to ‘give students more chances,’ but this can lead to poor outcomes as the committee can sometimes ‘get so stuck on that. We go down this path of let’s give them another chance’ (P6). Holistic principles may introduce bias (and compassion) into decision making, but for one member, operating with other principles may help mitigate professional bias to produce good outcomes. In their words: ‘I look more holistically…and take a step back and look at all of the repeated failures they’ve had, and then predict… “what are the chances that this person is actually going to be successful?” and that changes my view of it’ (P6).

Members share the expectation that collectively they will make decisions that result in good outcomes: Students whom they promote will be successful in clerkship. These predictions often require members to operate the model within their capacity as professional gatekeepers. As one member points out, they are members of a self-regulated profession and as such, they’ve ‘agreed to train those [who] come after [them] and part of that is evaluating and recognizing those who are not meeting [professional] standards, so ethically, [the Student Progress Committee] needs to be sure that [they’re] sending out learners better prepared’ (P8). This requires members to become more ‘hawk-like’ in their decision making which may be at odds with their ‘dove-like’ tendencies as educators. One member describes how they navigate these two conflicting roles to produce good outcomes:

I have very high expectations of the students. They’re going into a profession where people’s lives are at stake, and I think it’s important for us to be hawks …. I think, I may [also] have to be more “dove-like” [when] considering [student] circumstances…and [how] that may be impacting how they’re interacting with medical school. I think having that perspective…has been really useful and …helped me be more dove-like in my hawkishness (P8).

Members agree that a good outcome means supporting students for success. However, when decision making intersects with professional and pedagogical responsibilities, supporting a student for success might mean failing a student. As one member puts it:

We still need to make sure that we’re producing doctors that are safe, and that I have a responsibility to society, so I often remind myself of that as well, and that helps rejig my priorities…. I think it’s worthy to say, “no, you shouldn’t continue” (P6).

Members acknowledge the tensions that go into this type of high stakes decision making. One member states that ‘it’s that internal struggle of saying, “yes, there’s all these things going on, and I want to support them, and [be] fair,” but maybe part of that support means failing them and giving them a chance when the stakes are a bit lower’ (P8). While another member admits that ‘the whole thing is a tension, and it’s not comfortable …. I value and respect the importance of the work, but it’s not easy’ (P6).

## Discussion

Our findings contribute to an understanding of how one undergraduate CC operates within a shared mental model to produce reliable outcomes. In particular, it fills in a gap regarding how CCs perform their work, informed by members’ perceptions of different types of data to make judgements regarding student performance [22]. Results show tensions inherent within holistic decision making as some members feel comfortable with the subjective nature of the process while others perceive it to be less robust and prefer a more objective approach. However, holistic principles give structure to the decision-making process and having guidance and reminders during the meeting about how to implement these principles makes the process more robust and offers reassurance for its newer members.

Despite differences between postgraduate and undergraduate medical education with respect to the types of data inputs used in decision making, the perspectives expressed by the participants align with many of the recommendations for optimal functioning CCs based on the literature from postgraduate medical education. First, it aligns with recommendations for group composition [3,10] as its diverse membership was shown to generate a wide range of opinions and possible solutions allowing for flexibility in decision making but also as a mechanism to reduce member bias. Second, CCs members need to have a shared understanding of the group task [19,21,25] while not being inclined towards groupthink [36]. Groupthink is a negative outcome of team decision making as it introduces bias, but holistic principles may help mitigate this effect. Findings show that there is a high level of agreement (but not always total agreement) among members with respect to the final decision [19]. Members acknowledge that this is the outcome of a robust process that involves reflection, deliberation, openness, and having time for deliberation rather than one that relies on group conformity. Third, recommendations suggest open communication is essential for knowledge sharing [19,21,25] and that better and more defensible recommendations are made when information is shared within a structured decision-making process as it encourages discussion and invites the contribution of diverse opinions [7]. In our findings, Student Progress Committee members agree that one of the key principles of holistic decision making (and one of its challenges) is incorporating multiple sources of information including different members’ perspectives. This principle is facilitated by a structured communication that allows ample time for sharing and deliberation. Finally, robust decision making requires a series of checks and balances instilled within the process to ensure robust outcomes [10]. Members recognize the need to check their biases by balancing their judgements of student narratives against numerical data to minimize the potential to “fail-to -fail” students.

### Implications for practice and research

While we recognize the challenges of transferability to different groups and contexts, the shared mental model generated from the data suggests some guidance for other CCs in undergraduate medical education. We recommend that members of CCs need to be aware of the specific make-up of their shared mental model and how it operates within the local undergraduate context [19]. It is important to note any differences in the functioning of the model depending on certain characteristics such as the student’s level of education, the focus on formative assessments, and data used for assessment [19,24]. Moreover, our results suggest that faculty development is important to support CC members in making high-stakes decisions [37]. However, there is limited evidence to show what resources CCs need to perform their work, and whether the work of CCs varies across different specialities and programs [37] thus making it difficult to compare the undergraduate with the postgraduate context. Our findings may help fill in gaps about how institutions can support faculty development for CCs in both the undergraduate and postgraduate contexts. For example, it possibly could inform faculty development targets such as teaching how multiple raters and perspectives engaging in a qualitative, holistic review, leverages unavoidable subjectivity, while mitigating against bias. Transparently sharing the shared mental model construct, including localized findings, with new and existing committee members [18,19], provides a framework to help members conceptualize the ways they value different types of data, recognize their personal and professional biases, and understand how local medical culture and curricular structures influences decision making.

From a continuous quality improvement vantage point, seeking committee feedback on areas for data improvement within the model can help hone key-informant and data presentations that inform decisions. The shared mental model construct, and our unique findings, may be used to design interventions to enhance team performance or knowledge of holistic principles and to reduce bias. Uniquely positioned with a longitudinal holistic view, enriched by varied inputs and perspectives, CCs are not only charged with high-stakes decisions, but provide guidance to curriculum directors for interventions for students. Systematically leveraging the holistic view to identify areas for program improvement could be of great value for curricular, assessment, and student support imperatives in fostering tailored interventions for individual competency attainment within a fundamentally time-based program.

Research must continue to examine perspectives, including of learners, on which data inputs are necessary, and acceptable, in enhancing truly holistic decisions. Additional research could seek to further validate the shared mental model by examining whether committee decisions lead to positive impacts, not just in objective data, but in developing growth-oriented, adaptive learners. Additional research could focus on cross-institutional comparison of CCs to further extend our understanding of how local cultures and CC compositions are influencing decision making [19,38].

### Limitations

There are some notable limitations of our study. Foremost among them is situating the study in a single institution. As such, some of our findings will have limited transferability. However, this limitation is ameliorated by the detailed description we have provided of the research context and participant characteristics as well as the use of a well-established conceptual model which provides analytical tools that can help generalize our results. The shared mental model construct has been applied in assessment contexts in medical education and is a productive lens for understanding the activity of individuals as they examine and make decisions. At the same time, using one conceptual perspective to understand the activity of a complex social interaction such as a competency committee also poses drawbacks. Other conceptual orientations can provide alternative perspectives that complement, supplement, or contradict our account of CC activity [20]. *We do not claim that mental models are the only available perspective on CCs* and we hope other researchers will bring conceptual diversity as the field continues to investigate the role of CCs. Lastly, our study did not examine the more complex question of the efficacy and impact of CCs in undergraduate education. While this was beyond the scope of the current study, future work should examine this issue by examining longitudinal outcomes of CC decisions, both within, and beyond a training program.

## Conclusion

This study provides knowledge of how CCs operate at the undergraduate level to make informed decisions regarding student progress. A qualitative study using an abductive thematic analysis allowed for a conceptualization of a shared mental model that is unique to the Student Progress Committee and may offer other CCs at the undergraduate level a better understanding of decision making that is specific for their context. Members will have a better idea of what elements or inputs are used for decision making and how they can be manipulated to mitigate professional or personal biases. Although some members feel less confident with the subjective nature of holistic decision making, having a process based on principles gives it credibility for members who prefer a more objective approach. The results can inform the Student Progress Committee and guide its members in their quality improvement efforts and inform broader program-wide improvement while contributing more broadly to the literature on CCs, which is crucial given their role across the education continuum.

## Additional File

The additional file for this article can be found as follows:

10.5334/pme.1949.s1 Appendix. Tables 1 to 3.
