# Supplementary material for: Many Minds, One Model: Exploring Decision Making of an Undergraduate Medicine Competency Committee Using the Construct of a Shared Mental Model
Source: Perspect Med Educ. 2025 Aug 13;14(1):493–503. doi: 10.5334/pme.1949 (PMC12352382; doi:10.5334/pme.1949)
Supplement: Appendix. — Tables 1 to 3. [file pme-14-1-1949-s1.pdf]

Appendix

Table 1: Describing Elements or Inputs into Decision Making

**Theme**	**Subthemes**	**Codes**	**Description**	**Representative Quotes**
**Describing**	**Data**	Quantitative and Qualitative Data	Holistic decision making is a subjective or a contextualized process requiring input of both numerical data and student narratives.	**P7:** Just as important as [objective data] is student reflections on where they think they're at and how they might improve.<br>**P8:** The quantitative is really important… because…a pass at one percentage above the fail mark is very different than a pass comfortably in the eighties or nineties ….I wouldn't want to lose that quantitative.
		More data	Members identify that they need more longitudinal data to gain an overall holistic picture of student progress versus knowing what happened in just one course.	**P4:** For students who have been struggling over time, it's easier to understand [their] more longitudinal story…that would be helpful, that could be better.<br>**P5:** The robustness of our decisions could be improved by having more of that longitudinal data…as a snapshot.
		Precedent	Precedent is a source of data for decision making and members identify the need to treat "like cases alike," but they also recognize that holistic decision making requires individualizing of decisions.	**P7:** Every case is treated like a special case, and not one special case.<br>**P8:** We often talk about…precedent…but I don't know what precedent has been set. I don't know how [to] act in these circumstances [and] partly that's OK because we're supposed to be individualising our decisions.

		Perspectives of other members	The input of different member's perspectives offers multiple points of view which promotes discussion.	**P1:** You need a diverse committee that can offer lots of different viewpoints yet, paradoxically, you also need them to have at the end of the day a similar mental model of decision making.<br>**P8:** Differing opinions are…an attribute of the committee, as long as we can continue to engage in respectful disagreements.
		Veracity of student narratives	Incorporating student narratives is important for holistic decision making; however, some members are skeptical of the veracity of the narratives or their impact on student performance.	**P1:** Personally, I find narratives sometimes helpful, sometimes not, because I'm always wondering about the veracity of those narratives. I don't really have a legitimate reason to doubt them, but I have lots of experience in [education] and at the end of the day everyone will say very similar things.<br>**P3:** It felt like there were some people on the committee who were doubting the veracity, or the impact, the *true* impact of the systemic barriers that the [student] had [experienced] (emphasis, participant's).
	**Culture**			
		Professionalism (gatekeeping function)	Members are aware of their professional gatekeeping	**P5:** I have high expectations of the students. They're going into a profession where

			function which factors into their decision making.	people's lives are at stake, [so] … it's important for us to be hawks.<br>**P7:** On the one hand, I don't want to fail, but on the other hand, there are responsibilities to the profession and the public, maybe even the students themselves.
		Fail-to-Fail	Members agree that it is difficult to fail students, and the tendency is to give students the benefit of the doubt, but at the same time they recognize that professional standards must be maintained.	**P1:** We have a failure-to-fail culture in medical school that's well known, and part of the goals of the [SPC] is to change [that]. At the same time, it's still a high-stakes decision so people are reluctant to be heavy-handed.<br>**P7:** I find it very hard to fail students. We are looking at data that shows if they have failed or not, but…I would find it very hard to tell students they're not progressing.
	**Structure**			
		Diversity of Team	Members recognize that having a diverse committee can promote discussion and reduce bias in decision making.	**P5:** I think it's important to have a diversity of opinions. It would be really useful as well [to have] diversity based on gender [or]…racial diversity.<br>**P7:** We have…diversity in practice…. The roles within medical education are different, people have different experiences through different roles with students.

	**Biases**			
		Personal and professional biases	Members have personal and professional biases which can inform decision making, but paradoxically, it may also bias decision making.	**P1:** The advantage I bring is having a biased perspective. People are there for their subjectivity, or their perspective; it's important to bring that out.<br>**P5:** You come with your own personal bias [whether] you can relate [or not] to the student that's being discussed…. There's your role as an educator: if you're in charge of assessment, you will have a different perspective than someone else whose role is focused on equity.
		Biased data	Members recognize that NVMs who present student narratives may be biased in their telling of the narrative which can sway decision making.	**P6:** I think they have a chance for success because if the [NVM] says that then the committee is more likely to vote in favour of giving the student a rewrite.<br>**P8:** I also recognize the bias that comes with it because there's someone who's presenting a very compelling story about a learner…but it is still helpful to have that information.
		Biased or preferred approach to decision making	Holistic decision making may be new for some members who prefer a more algorithmic approach. Part of	**P5**: I like certainty and consistency in [my] approach and so I wanted to apply [an] …algorithm to each of the cases …. I still have that preference, but over

			the learning curve for new members is becoming more comfortable with a subjective process.	time…, I take into account other data that is not linked to the assessment itself, but provides context on the student and their particular case.<br>**P7:** I try to gather as much information as possible as I would make any decision…. I try to be as objective as possible recognizing that there's always some bias that comes into it, and trying to be aware of that bias as well.

Abbreviations: NVMs= Non-Voting Members; SPC= Student Progress Committee;

Table 2: Explaining or Comprehending Meaning of Model Elements

Theme	Subthemes	Codes	Description	Representative Quotes
**Explaining**	**Processes**	Balancing	Members recognize that holistic decision making requires balancing not only each other's perspectives but also their roles as educators and professional gatekeepers.	**P5:** It comes from the perspective of what's in the best interest of the students, and how do we…best support [students] for success…, but also keeping in mind, how do we ensure that standards and expectations around performance are also met. So, I feel like those two really go hand in hand. **P8**: We all know each other's roles, and as you work together as a committee, you recognize that I'm going to bring one perspective and there's always this one person who brings their other perspective and it is sort of that balancing of perspectives.
		Judging	Holistic decision making requires weighing-in on multiple data points including making judgements on student narratives and whether they are compelling enough to grant reassessment.	**P5:** There are times when…I don't see [the narrative] as [being a] compelling enough reason, especially… if there's been a pattern of challenges with assessments [or] not meeting standard over time…that's where I struggle. **P6:** It's a constant negotiation of what is the data, [and] how do you interpret the data,

				[and] where are you putting your weight as to what is important.
		Shifting perspectives	Members recognize that it is important to be challenged in their decision making and shifting one's perspective requires being open to different opinions.	**P5:** [I've] shifted my perspective and it's definitely happened in meetings where I've felt a certain way, but other information provided by group members has helped me think differently. **P7:** Shifting perspectives is always an important part of being in a group. At first, I would just listen because I wanted to make sure that my contributions were valuable. I really listen to different perspectives. I still do.
		Reflecting	Reflecting is an important part of the process and is considered to be a key principle of holistic decision making.	**P2:** If [students don't take advice] that's a concern, and you start to say to yourself, "will this person succeed going forward?", so [that's]…what I am really listening for. **P7:** I think the only strategy is to be aware of bias, to self-reflect [as] some cases resonate more with me than others.
		Listening	Listening is an important part of the process and is considered to be a key principle of holistic decision making. It allows for the input of multiple data	**P4:** I listen carefully to all of the insights and wisdom that are being brought about the experiences of these students in this broader context, and try to participate in arriving at a shared decision

			points including student narratives and the opinions of other members.	about what is best and most accountable for how to deal with the difficult situation of these students not meeting the bar.<br>**P8:** The committee members [are] listening to whoever is presenting the learner story, so there's a fair amount of onus on the [NVM] to actually have done their due diligence [regarding] how they've presented the case because that's where we listen for the differences.
		Negotiating	Negotiation is an important part of the process and is a key principle of holistic decision making.<br>Allocating time in the meeting for discussion gives the process its integrity.	**P5:** I don't really see it as a negotiation around decision making, I see it more as like a shared discussion, more of a dialogue.<br>**P7:** I think that's the point of the Progress Committee. If there was no discussion it would just be based on grades.
	**Structural**	Reminding	Having repeated reminders in the meeting from the chair about the structure and function of the committee helps keep members on track.	**P6:** We're supposed to take a holistic view of the student and … that would be like an important principle for us to have more guidance on…. Reminders about that from the committee chairs would be helpful.<br>**P7:** The committee is very diverse in terms of its members so if one

				person doesn't have a value we are reminded of that value.

Abbreviations: NVMs= Non-Voting Members

Table 3: Predicting or Outcomes of Decision Making

**Theme**	**Subtheme**	**Codes**	**Description**	**Representative Quotes**
**Predicting**	**Likelihood of Success**		Members predict the likelihood of student success through the process of holistic decision making. Members reflect on the consequences of making poor predictions.	**P2:** I try to be careful to look for other sources of data that support my view that this person is not going to be successful moving forward, …and [I'm] not just reacting to their resistance. **P5**: For students who have not met with the course director, or…handed in their focused learning plans on time…I worry about the future success of that student--and it is a profession.
	**Support and Success**		Members will support students for success when there is a standard in place.	**P5:** I'd be more compelled to support someone…if there's a story of them being engaged…in [their] improvement. **P7:** It's so important to support students in their remediation, and to give them that opportunity to redo things instead of just passing them along.

	**Good Outcome/Bad Outcome**		Good outcomes are framed as promoting students whom members feel will be successful.	**P7:** A good outcome is success, right?.... True success (from a MD's perspective) would be diagnosing the learner, prescribing a management plan and then seeing success as a result of that, just like a patient getting better.
			Good outcomes are a result of members predicting which students will struggle later on in clerkship.<br><br>A bad outcome would be to continually pass students who are not meeting standard and whom members predict will struggle in the future.	**P8:** If the SPC has done its job well, learners are identified who need remediation in the pre-clerkship years, so they should not end up failing in clerkship.

Abbreviation: SPC= Student Progress Committee
